# Supplementary figures and images for: Selectivity profiles and substrate recognition of Rab-phosphorylating kinases
Source: Biochem J. 2025 Sep 4;482(17):1307–19. doi: 10.1042/BCJ20253212 (PMC12599248; doi:10.1042/BCJ20253212)

LRRK1

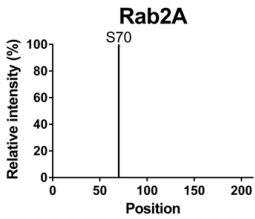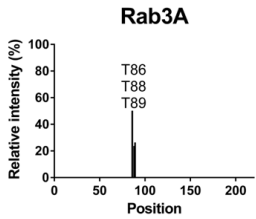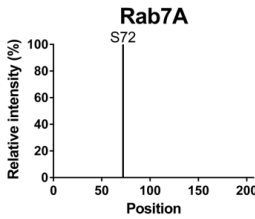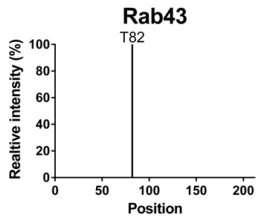

LRRK2

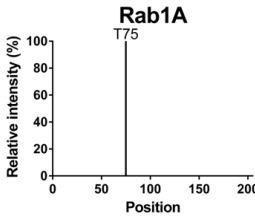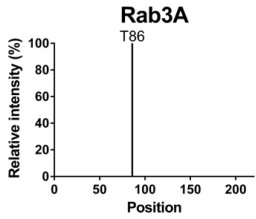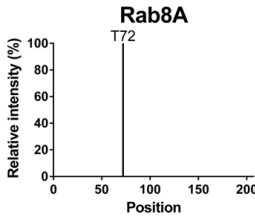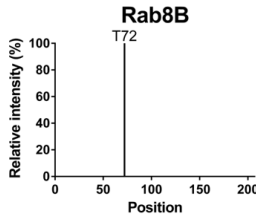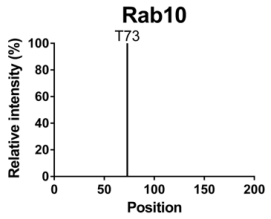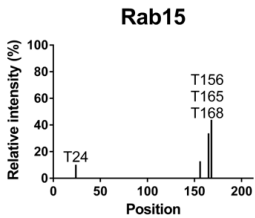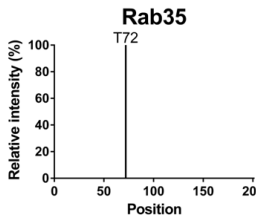

DYRK1

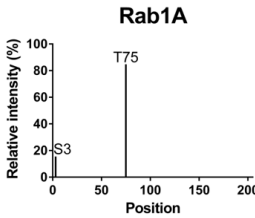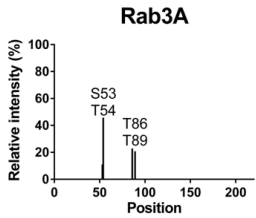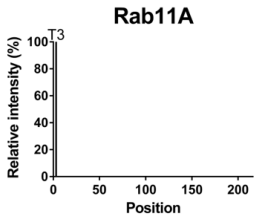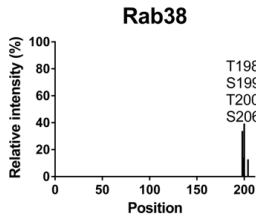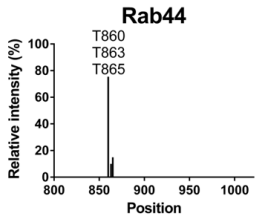

MST1

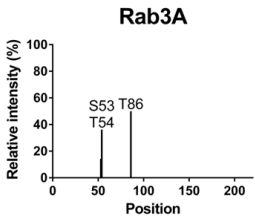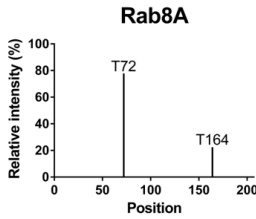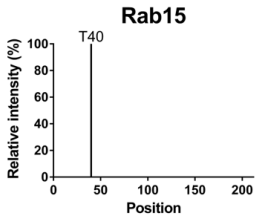

TBK1

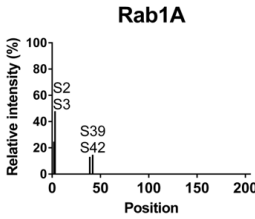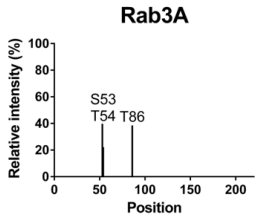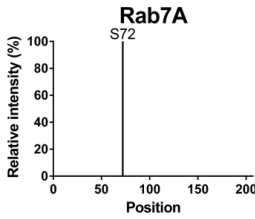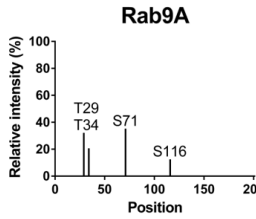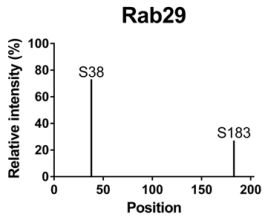

Supplement: Supplementary Figure S1 [file bcj-482-17-BCJ20253212-s001.pdf]

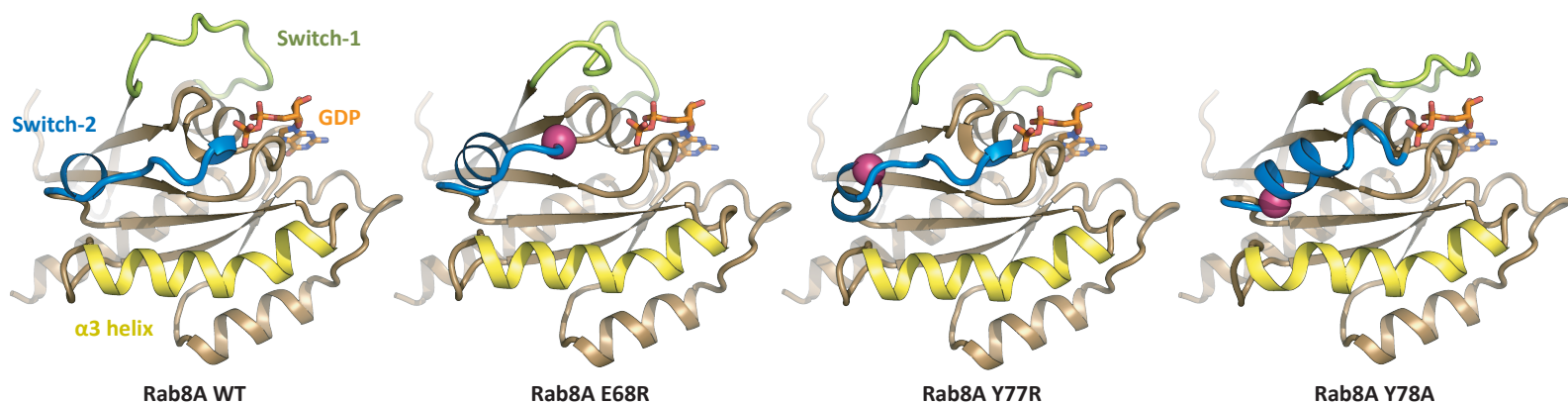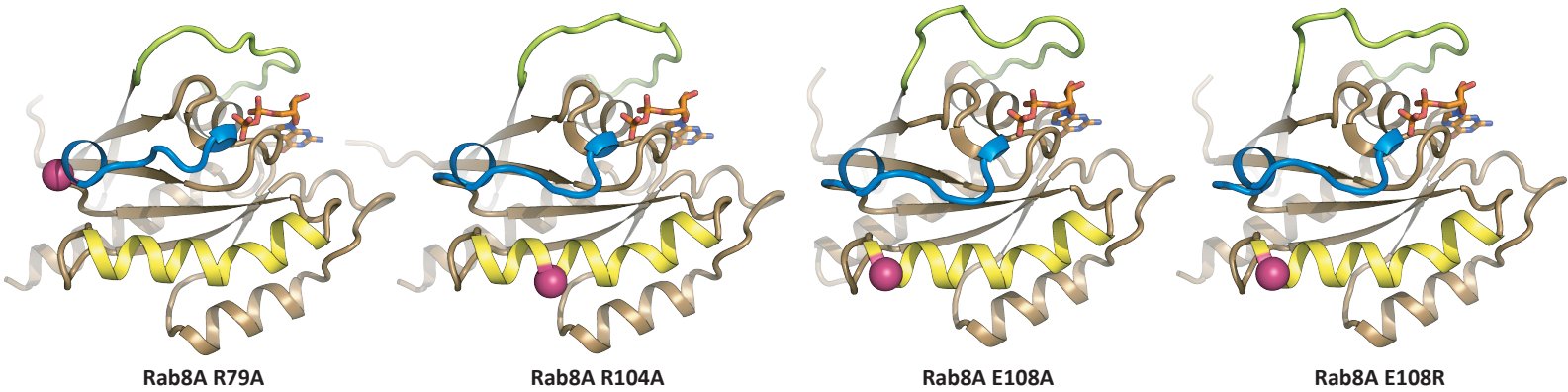

Supplement: Supplementary Figure S2 [file bcj-482-17-BCJ20253212-s002.pdf]

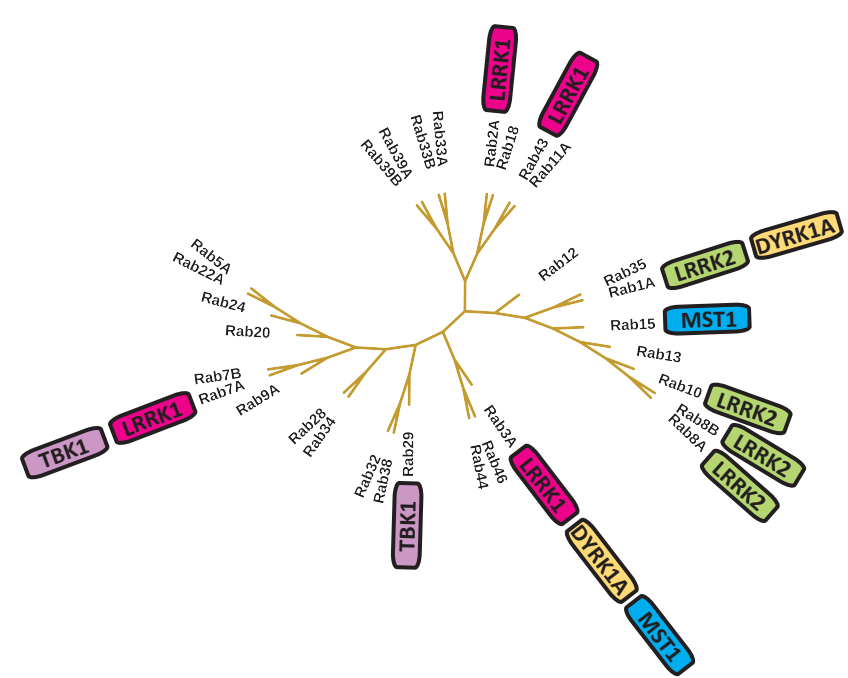

Supplement: Supplementary Figure S3 [file bcj-482-17-BCJ20253212-s003.pdf]
